# Correlation of adverse effects of cisplatin administration in patients affected by solid tumours: A retrospective evaluation

**DOI:** 10.3892/or.2013.2279

**Published:** 2013-02-06

**Authors:** LAURA ASTOLFI, SARA GHISELLI, VALERIA GUARAN, MILVIA CHICCA, EDI SIMONI, ELENA OLIVETTO, GIORGIO LELLI, ALESSANDRO MARTINI

**Affiliations:** 1 Bioacoustics Research Laboratory, University of Padua, I-35129 Padua; 2 ENT Surgery, Department of Neurosciences, University of Padua, I-35129 Padua; 3 Department of Life Sciences and Biotechnology, University of Ferrara, I-44121 Ferrara; 4 Clinical Oncology Unit, St. Anna University-Hospital, I-44121 Ferrara, Italy

**Keywords:** adverse effect correlation, cisplatin toxicity, solid tumours, chemotherapy

## Abstract

Cisplatin is the most common antineoplastic drug used for the therapy of solid tumours. To date, researchers have focused on the dosage to be administered for each specific tumour, mainly considering the local adverse effects. The aim of this study was to correlate the severity of the adverse effects with: i) the dosage of cisplatin; ii) the specific site of the tumour; iii) the association with other drugs; and iv) the symptoms. We analysed data from 123 patients with 11 different tumour classes undergoing therapy from 2007 to 2008 at St. Anna Hospital (Ferrara, Italy), using the Spearman non-parametric correlation index. Even though significant correlations were found among the variables, the overall results showed that the main factor influencing the severity of the adverse effects was the dosage of cisplatin administered.

## Introduction

The majority of therapies for malignant tumours are based on chemotherapeutic drugs with cytotoxic effects, which cause death of tumour cells by direct damage to DNA or by inhibition of cell division. Unfortunately, these drugs are mostly unspecific, therefore, their administration often causes extended tissue toxicity (1).

Cisplatin, or *cis*-diamminedichloroplatinum(II) (CDDP) was the first platinum-based anticancer drug developed for clinical purposes. It is employed in children for treatment of haematological tumours and in adults for treatment of solid tumours such as testicular (2), prostatic and ovarian (3,4), uterine, cervix, breast, bladder, stomach (5,6) colon, brain, head-neck (7,8) and both non-small and small-cell lung cancer (9,10).

The intravenous administration of cisplatin causes an increase in initial tissue accumulation and in plasma levels for an extended time. Cisplatin levels are high in plasma as the drug reversibly binds to >90% of proteins; traces of the drug are often detected years after chemotherapy (11,12). Brouwers and co-researchers (13), during a 6-year follow-up screening, demonstrated that plasma levels of cisplatin have a half life (t/2) of 28.5 months (14,15). Moreover, the elimination of the drug is not the same in all tissues; elimination is faster in more rapidly regenerating tissues in comparison to slower ones and each tissue has its own t/2 (16). The plasma levels of cisplatin depend upon several factors: cumulative dose, follow-up time, age of patient, glomerular filtration speed (GFR) during chemotherapy, use of sodium thiosulphate (STS) during cisplatin chemotherapy and method of administration (11,13). A higher initial dose of the drug causes a higher tissue concentration and a longer time for excretion, yet STS binding to cisplatin lowers its initial tissue accumulation (16).

Cisplatin has both biliary (~10%) and urinary (90%) clearance. The urinary clearance of cisplatin is characterised by an initial fast excretion phase (20 min), followed by a second slower phase (60–70 min) and a third very slow and incomplete phase (24 h) (17,18).

The activity of cisplatin and the appearance of the side effects depend on pharmacological parameters such as the dosage (single and cumulative) and administration (schedule and means), but also on systemic and individual conditions such as skin pigmentation, age, diet, blood pH and interactions with radiotherapy (19–21). Certain effects are dose-dependent; thus, they can be controlled but not prevented (22–24).

Among the adverse effects that may develop, the most frequent ones are gastrointestinal symptoms. More than 90% of patients experience nausea and vomiting; these symptoms are counteracted by the administration of antiemetic drugs such as the antagonist of serotonin receptor 3 (5-HT_3_) and dexamethasone. In a smaller number of cases, general symptoms are detected, such as fever, hyposthenia, altered sleep-wake cycle, myelosuppression and alteration in the liver, skin and respiratory apparatus. Among the negative side effects with a more or less severe involvement of tissues include neurotoxicity, nephrotoxicity and ototoxicity (2,13,25–27).

The different responses to cisplatin treatment depend on individual factors and on resistance mechanisms. Resistance mechanisms include: reduction in intracellular drug accumulation, drug inactivation by the cytosol, changes in DNA repair mechanisms and alterations in proteins involved in apoptosis (18,28–31). Resistance may be induced by only one or several of the above mentioned mechanisms (31). The reduction in intracellular accumulation may depend upon an increased drug outflow or a reduced inflow through the cell membrane. The inflow by membrane channels may be modified by several compounds including amphotericin B, aldehydes, inhibitors of Na-K adenosine triphosphatase and ouabain (18). Molecules such as glutathione and metallothioneins may react with cisplatin at the intracellular level, inactivating it and preventing its binding to DNA. Others may reduce its chemotherapeutic efficacy by modifying the DNA repair mechanisms (32); among these there are the DNA polymerase inhibitors (zidovudine and ganciclovir), the inhibitors of topoisomerase II (etoposide and novobiocin), the methylxanthines (caffeine and pentoxifylline) and specific chemotherapeutic drugs (5-fluorouracil, cytarabine and hydroxyurea) (33,34).

The different compounds studied for interference with cisplatin toxicity inlcude sodium thiosulphate, D-methionine, vitamins C and E, *Gingko biloba* extract and dexamethasone (25,35–38).

To date, most clinical literature data report that the efficacy and side effects of cisplatin treatment are associated with other compounds in patients belonging to homogeneous groups or with tumours in specific body sites. The purpose of the present research was to evaluate the incidence of side effects in patients with different types of tumours, undergoing chemotherapy with cisplatin. Records of the patients retrospectively examined were heterogeneous, in order to verify i) whether various chemotherapy combinations increase the sensitivity of the organism to the toxic effects of the drug; ii) whether a direct correlation could be detected between the tumour site and a specific side effect; and finally iii) whether the side effects were reciprocally related. For this purpose, we examined the medical records of 123 patients treated with cisplatin in the same hospital (St. Anna University Hospital, Ferrara, Italy) during 2007 and 2008, with special attention to the dosages and side effects reported.

## Materials and methods

### Study population

The medical records of 123 patients (81 males and 42 females), undergoing chemotherapy during 2007 and 2008 at the Clinical Oncology Unit, St. Anna University Hospital in Ferrara (Italy), were retrospectively examined in agreement with Italian privacy and sensitive data laws (D.Lgs 196/03) and according to the institutional guidelines of the St. Anna University Hospital.

### Tumour distribution

All malignant tumours were classified according to the Italian Association of Cancer Registries (AIRTUM, Associazione Italiana Registri Tumori) and the International Classification of Diseases. The cancers were recognised as follows: lung, head and neck, gynaecological, melanoma, thymoma, gastric, occult, neuroendocrine, urothelial, hepatic and thyroid.

### Treatment

Doses and methods of cisplatin treatment were modulated according to the drug therapeutic plan (alone, in association with other chemotherapeutic agents or with radiotherapy), depending on the tumour type and on the conditions of the patients.

Cisplatin (*cis*-diamminedichloroplatinum(II), CDDP) was administered alone or with gemcitabine (GEM), epirubicin (EPI), etoposide (VP-16), 5-fluorouracil (5-FU), dacarbazine (DTIC), vinorelbine (VNR) or in a combination called EDOC (EPI + CDDP + vincristine + cyclophosphamide). In all cases, the drug treatment was preceded by hydration and by antiemetic treatment with dexamethasone and serotonin (5-hydroxytriptamine 3, 5-HT_3_) (from 30 min to 1 h and 30 min before chemotherapy). The pretreatment was recommended by the American Society of Clinical Oncology (ASCO, 2006) since cisplatin is one of the chemotherapeutic agents with the most severe emetic side effects (incidence >90%). Although the daily standard dose of dexamethasone is 20 mg, in most cases the prescribed daily dose was 8 mg in 100 ml of saline solution, administered intravenously. The 5-HT_3_ drugs are a group of antagonists of the 5-HT_3_ serotonin receptor (ondansetron, granisetron or dolasetron). The method of administration (oral or intravenous) does not influence their efficacy in controlling symptoms. The administration of the chemotherapeutic drugs was also preceded by administration of two diuretics (furosemide and mannitol). The hydration of the patients undergoing chemotherapy with cisplatin is necessary to reduce dehydration and the relevant nephrotoxic effects of the drug. When cisplatin is administered with GEM, 5-FU or VNR, additional administration of dexamethasone is required (a total of 16 mg), and when the therapy follows the EDOC scheme, the amount of serotonin is doubled as well. Among the 123 patients studied, 63 were also treated by radiotherapy, particularly when affected by head-neck tumours.

### Classification of adverse effects

The side effects observed, following the Common Terminology Criteria for Adverse Events (CTCAE) v3.0 (National Cancer Institute, 2006) were respectively categorised as follows: auditory/ear (ototoxicity), blood/bone marrow (haematological toxicity), constitutional symptoms, dermatology/skin (dermatological disorders), gastrointestinal (gastrointestinal disorders), hepatobiliary/pancreas (hepatic toxicity), neurology (neurotoxicity), pulmonary/upper respiratory (respiratory disorders), renal/genitourinary (nephrotoxicity) and sexual/reproductive function (genital apparatus disorders). Changes in sleep-wake cycle were classified in a separate category, termed sleep-wake disorders, as they are not clearly categorised by CTCAE.

### Statistical analysis

The collected data represent cancer prevalence in 2008 among oncological patients of the St. Anna University Hospital of Ferrara, undergoing therapy for a maximum of 6 years.

For all data, the average values and standard deviations were calculated for dosages and the frequency of side effects detected in all patients and for patients grouped by tumour type. Concerning the possible association between the examined variables, we calculated the Spearman non-parametric correlation. The data were verified by r-Pearson and were plotted in dendrograms by the unweighted pair-group method using arithmetic averages. Statistica 7 (StatSoft srl., Italy, 2005) software was used.

## Results

### Patient characteristics

The patients had a mean age of 60.0±9.9 (SD) years (calculated when they received the first treatment with cisplatin) and an age range of 35–81 years. An overview of the data is shown in Table I, which reports the number of patients by gender, years since diagnosis and type of cancer.

Concerning the incidence of tumours in relation to gender, we calculated the relative percentage for each tumour typology. Thyroid and obviously gynaecological tumours were restricted to females, while gastric carcinoma was detected only in males. A frequency of 50–60% was detected in occult carcinoma, hepatic carcinoma and melanoma (one male patient had hepatic carcinoma and one female had biliary tract cancer). In all other tumours examined the incidence was higher in males in comparison to females.

As shown in Table I, the tumours with the highest prevalence were those of the lung and head and neck, with a 4:1 male:female ratio. The third most frequent tumours in women were gynaecological tumours. In all other cases, the number of affected individuals ranged from 2 (occult, hepatic and thyroid cancers) to a maximum of 5 (melanoma). In Table I different tumour histotypes were grouped together. For example, all female tumours (5 cases of cervical cancer, 4 of ovarian cancer, 4 of breast cancer, 3 of endometrial cancer, one of breast-endometrial cancer and one of vulvar cancer) were grouped under the GN (gynaecological) class. Lung cancers [42 non-small cell lung cancer (NSCLC), 9 small cell lung cancer (SCLC), one sarcomatoid carcinoma and two unspecified cases] were grouped under the L (lung) class.

### Recording of adverse effects

The majority of patients had gastrointestinal disorders, such as nausea and vomiting, diarrhoea, constipation, epigastralgia, pyrosis, dysphasia, postprandial abdominal bloating sensation, white tongue, dysgeusia and taste impairment. Among the other adverse effects, constitutional symptoms included hyposthenia and asthenia, fever, weight loss and appetite loss. Cisplatin myelosuppression caused haematological toxic effects, such as anaemia, leukopenia, neutropenia and thrombocytopenia. The dermatological disorders included alopecia, itchiness, skin rash, edema, arm phlebitis and mucositis. Neurotoxicity mainly involved the peripheral system in comparison to the central nervous system. The most common symptoms were paraesthesia, followed by cephalalgia, speech impairments, aphasia, agnosia, lipothymia (near-fainting syndrome), convulsions, panic and transient ischemic attacks (mini-strokes), visual failure, sensory-motor deficits and motor coordination impairments. Nephrotoxicity included electrolyte alterations (hyperK^+^, hypoK^+^, hyperCa^++^, hypoNa^+^, hypoCl^-^, hypoMg^+^), an increase in blood nitrogen, creatinine and urea, pollakiuria (abnormally frequent urination), hematuria, oliguria, polyuria, urinary tract infections, and kidney spasms to renal insufficiency. Hepatic toxicity was characterised by hepatomegaly and a rise in hepatic enzymes (transaminases, bilirubin, γ-glutamyl transpeptidases). Respiratory disorders mostly involved cough, dyspnea, polypnea and chest pain. Symptoms of ototoxicity mainly included vertigo, in a few subjects tinnitus or hypoacousia. Genital apparatus disorders included female gynaecological symptoms such as vaginal discharge.

### Chemotherapy protocols and adverse effects

Analysis of the average daily doses administered revealed that the most intensive therapy (the highest dosage) was applied for lung sarcoma tumours (treated with 148 mg/m^2^ of the chemotherapeutic drugs), followed by SCLC (143.75 mg/m^2^) and neuroendocrine tumours (136.6 mg/m^2^). The lowest daily dosage was administered to patients affected by thyroid tumours (45 mg/m^2^), followed by patients affected by uterine and vulvar tumours (54 mg/m^2^). The patients receiving a higher CDDP dosage were affected by neuroendocrine tumours (cumulative dose 635.19 mg/m^2^), followed by those affected by urothelial tumours (621.81 mg/m^2^) and SCLC (503.125 mg/m^2^). The patients receiving the lowest cumulative doses (69 mg/m^2^) were those affected by ovarian tumours. Finally, doses <100 mg/m^2^ were administered to the patients with uterine, gastric, vulvar and thyroid tumours.

**Figure 1:**
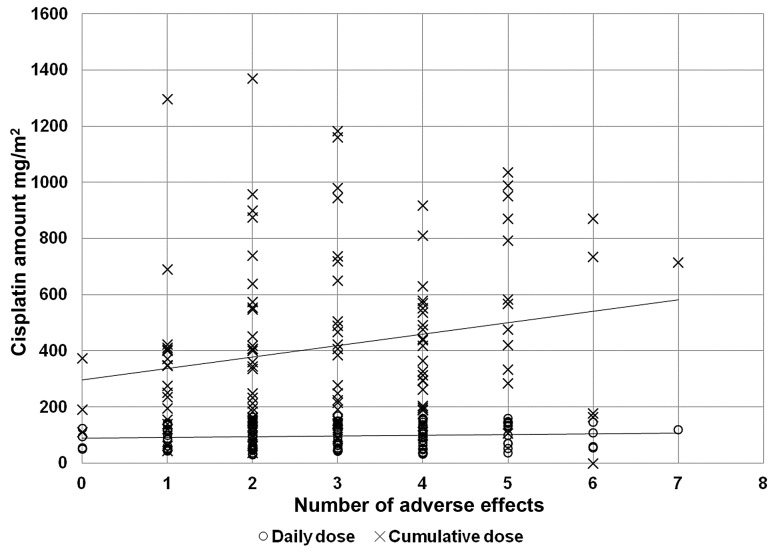
Correlation between administered drug amounts and number of adverse effects. The daily and cumulative cisplatin amounts are expressed in mg/m^2^.

In order to verify the correlation between the chemotherapy dosage and the incidence of adverse effects, we plotted the number of toxic effects against cumulative and daily doses of cisplatin administered (Fig. 1), and we correlated these doses with the associated compound to the adverse effects (Table II). As expected, the cumulative amount of cisplatin was directly related to the number of adverse effects (r^2^=0.3826, P<0.001). The daily dose correlated with gastrointestinal and respiratory disorders, while the cumulative dose also affected the hepatic and haematological systems.

### Tumour site and adverse effects

To study in more detail the side effects detected in the different tumour classes, the number of subjects were determined exhibiting each type of side effect ordered according to tumour class (Table II). No side effect was common to all tumour classes, and the most frequent side effects were gastrointestinal toxicity, constitutional symptoms and haematological toxicity. The constitutional symptoms and gastric toxicity were consistently detected, except in patients with thyroid tumours. Apart from systemic and gastric symptoms, the patient with hepatic tumours also showed dermatological alterations, while patients with lung cancer presented all side effects together. The less common symptoms detected were sleep-wake disorders and alterations of the reproductive, respiratory and auditive tract. The less frequent side effect was genital apparatus toxicity. Following analysis of the number of side effects detected for each tumour class, it was possible to note that the toxicity range was higher in individuals affected by lung cancer, while in those affected by thyroid and hepatic cancer the number of side effects was lower. The effects of chemotherapeutic associations were analysed by Spearman non-parametric correlation. The statistically significant results are reported by tumour and by year of diagnosis in Table III. No correlations were found among chemotherapeutic protocols and adverse effects in thymoma, gastric, occult, neuroendocrine, urothelial, hepatic and thyroid tumours.

### Side effects are reciprocally related

**Figure 2:**
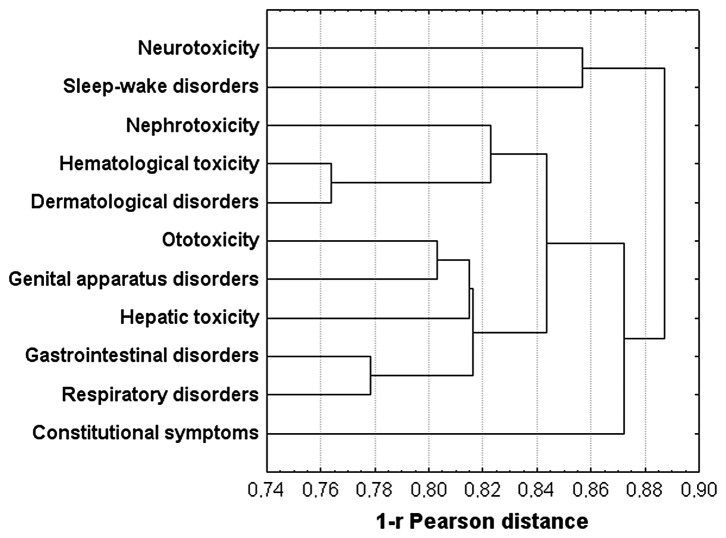
Correlations among side effects calculated by 1-r Pearson distance and plotted according to hierarchical union rank.

Based on the differences in side effects among the tumour classes, we also examined the possible association of side effects together or to any tumour class by Spearman non-parametric correlation. The results, summarised in Table IV, support the hypothesis that dermatological disorders were associated with sleep-wake disorders and haematological toxicity, gastrointestinal disorders were associated with respiratory disorders and ototoxicity, and genital apparatus disorders were associated with ototoxicity and hepatic toxicity. The last result was also found in the dendrogram of the data plotted using the hierarchical union method (Fig. 2). Notably, neurotoxicity was associated with sleep-wake disorders and, together with constitutional symptoms, stand out from all other groups.

## Discussion

The present study describes the frequency of side effects in 123 patients affected by solid tumours and treated with cisplatin at the St. Anna Hospital of Ferrara, Italy during 2007 and 2008. This is the first study considering heterogeneous populations as previous literature data concern studies limited to specific tumours (4,39–43).

The highest tumour incidence was in males (63%), and the most frequent tumour was lung tumour (43.4%, with a male/female 4:1 ratio and average age of 62 years), as supported by data from Regione Emilia-Romagna (Italy) reported in 2006 (44).

The most frequent adverse effects detected (~72%) involved the gastrointestinal apparatus and constitutional symptoms (fever, hyposthenia, asthenia, weight loss and sleep-wake rhythm alterations) while the least frequent effects involved genital apparatus disorders (2%). The highest number of side effects was detected in patients with lung cancer; this could be due to the fact that this was the most frequent tumour class. To attenuate the adverse effects of cisplatin treatment, it is necessary to evaluate individual variations due to age, duration of therapy, range of doses and synergism with other compounds causing the toxicity. Considering the therapeutical protocols associated with different tumours, we examined the cisplatin dosages administered. According to this analysis, no direct correlation was noted between the administered daily doses of cisplatin and the other chemotherapeutic drugs involved. During chemotherapy, dexamethasone is applied as an antiemetic (45) and only recently its otoprotective qualities have been recognised in animal models (37). Correlation analyses showed that dexamethasone protects against nephrotoxicity in lung tumour patients. However, a positive correlation between the drug dosage and neurotoxicity was detected in both lung and head-neck tumour patients. In esophageal tumours, the rate of successful cisplatin treatment is 25–35% for metastatic carcinomas and 50–60% for local tumours at an advanced stage (18). For head and neck tumours, cisplatin is usually administered in association with 5-fluorouracil and radiotherapy (7,18). In our data set, radiotherapy was inversely correlated to constitutional symptoms, thus, showing a protective effect. In lung tumour patients, the cisplatin dosage was the main cause of adverse effects. However, in NSCLC, cisplatin-based chemotherapy shows a complete response only in ~30% of cases; therefore, it represents only a palliative care treatment (40). Cisplatin is usually administered in association with GEM at the suggested weekly dose of 51 mg/m^2^ (with gemcitabine 1500 mg/m^2^) (40). In SCLC, cisplatin is usually administered with VP-16; if the tumour is locally advanced, the positive response to chemotherapy is ~50–60%, with an average survival time of 7–11 months. In more advanced tumours, paclitaxel is administered with cisplatin, with a positive response rate of ~34–41%. After chemotherapy, the relapse of tumours occurs in 95% of cases (9,31). For melanoma treatment, radiotherapy is correlated with the increasing number of adverse effects and decreased cisplatin dosages are associated with constitutional symptoms. The literature data report that cisplatin is administered alone or with VNR and DTIC, at doses ~100–200 mg/m^2^. The positive response is ~16% (46). In patients with gynaecological tumours, the cisplatin dosage was not correlated with any adverse effect; in cervical cancer the chemotherapy with cisplatin (not associated with other drugs) usually follows surgical and radiotherapy treatment. Among patients with ovarian cancer, 70% show an initial positive response to cisplatin but only <25% survive up to 5 years. In these tumours, cisplatin is employed in association with paclitaxel or topotecan (3). In the present study group, paclitaxel was correlated with both neurotoxic and nephrotoxic effects. Concerning the other tumour classes, no correlations with adverse effects were detected. In urothelial (or transitional cell) carcinomas, locally advanced or metastatic, and particularly in bladder cancer, cisplatin has been recommended for the last 20 years in association with methotrexate, vinblastine and doxorubicin (M-VAC). This drug combination yielded positive results in 50–70% of cases, with a 10–20% complete response and an average survival time of ~1 year (18). However, the high toxicity of this drug combination promoted successful research on new combinations of compounds, such as gemcitabine, taxane and paclitaxel. Cisplatin combined with gemcitabine and paclitaxel yielded a positive response in 78% of cases, with an average survival time of 24 month (47). Thymus tumours (including thymoma) at stage III or IV are routinely treated by polychemotherapy mainly involving cisplatin, adriamycin, etoposide, cyclophosphamide or ifosfamide (48,49).

After verifying the most common side effects and which tumour class showed the highest frequency, we also examined the possible associations among the different side effects. The results of these analyses, performed using the Spearman non-parametric correlation test, showed a close relationship between dermatological disorders and either haematological toxicity or sleep-wake disorders, and between respiratory and gastrointestinal disorders, as previously shown in the literature (50).

Other correlations were found between genital apparatus disorders and either ototoxicity or hepatic toxicity, and between ototoxicity and gastrointestinal disorders. These relationships may be due to metabolic effects, but they are difficult to explain as the patient group was highly heterogeneous. Moreover, gastrointestinal disorders are the most common side effects in patients undergoing chemotherapy (50). Ototoxicity is mostly detected in patients with lung or head and neck tumours treated with a high dosage of cisplatin.

In conclusion, through analysis of this heterogeneous group of patients, we confirmed that the main factor influencing the occurrence and severity of adverse effects is the dosage of cisplatin administered, both for single and cumulative doses.

## Figures and Tables

**Table I:** Patient characteristics.

Gender	Years since diagnosis	Age ± SD	Tumour classes										

			L	HN	GN	M	TM	GS	O	N	U	HE	TY
Male													
	1	61±9.9	14	7	-	1	-	2	1	1	1	-	-
	2	62±8.2	23	9	-	1	2	1	-	-	1	-	-
	3	60±9.2	6	5	-	1	-	-	-	1	-	1	-
	4	66±13.4	2	-	-	-	-	-	-	-	-	-	-
	6	39	-	1	-	-	-	-	-	-	-	-	-
Female													
	1	56±11.5	1	1	5	1	-	-	-	1	-	-	-
	2	60±13.4	3	4	5	1	-	-	1	-	-	-	2
	3	58±8.3	4	1	7	-	1	-	-	-	1	1	-
	4	53±5.7	1	-	1	-	-	-	-	-	-	-	-

Number and mean age (± standard deviation) (in years) of oncological patients grouped according to gender, years since diagnosis and tumour class. L, lung; HN, head and neck; GN, gynaecological; M, melanoma; TM, thymoma; GS, gastric; O, occult; N, neuroendocrine; U, urothelial; HE, hepatic; TY, thyroid.

**Table II:** Chemotherapy protocols and adverse effects.

	Cisplatin dose		Tumour classes													
					
Adverse effects	Day	Cum	L	GN	U	GS	HE	N	TM	HN	M	TY	O	Total	AE (%)	S (%)
Constitutional symptoms	0.274	0.130	47	10	4	2	1	2	3	14	4	0	1	88	23	72
Dermatological disorders	0.500	0.860	15	3	2	2	1	2	0	15	3	0	0	43	11	35
Gastrointestinal disorders	**0.009**	**0.000**	45	14	4	2	1	3	2	13	4	0	1	89	24	72
Genital apparatus disorders	0.596	0.365	1	0	0	0	0	0	1	0	0	0	0	2	1	2
Haematological toxicity	0.338	**0.001**	29	7	2	2	0	2	3	14	3	2	2	66	18	54
Hepatic toxicity	0.151	**0.009**	9	2	0	0	0	1	0	1	0	0	0	13	3	11
Nephrotoxicity	0.264	0.950	10	3	1	0	0	1	2	2	1	1	0	21	6	17
Neurotoxicity	0.126	0.269	14	3	4	1	0	2	0	4	2	1	1	32	9	26
Ototoxicity	0.651	0.211	4	2	2	0	0	0	0	3	0	0	0	11	3	9
Respiratory disorders	**0.021**	**0.038**	5	2	1	0	0	1	0	1	0	0	0	10	3	8
Sleep-wake disorders	0.488	0.915	5	0	0	0	0	0	0	0	1	1	0	7	2	6

Spearman non-parametric correlation between daily or cumulative cisplatin dosage and adverse effects, and the number of oncological patients showing the different toxic effects (rows) subgrouped according to the different tumour classes (columns), identified according to the National Cancer Institute Guidelines (2006). Day, cisplatin daily dose; cum, cisplatin cumulative dose; L, lung; GN, gynaecological; U, urothelial; GS, gastric; HE, hepatic; N, neuroendocrine; TM, thymoma; HN, head and neck; M, melanoma; TY, thyroid; O, occult; AE%, percentage of adverse effects; S%, percentage of patients affected. Bold print indicates statistical significance; P<0.05 significant, P<0.001 highly significant.

**Table III:** Tumour site and adverse effects.

Adverse effects	Treatment	SpR	t(N-2)	P-value
Gynaecological tumours				
Respiratory disorders	EPI	0.686	3.771	0.002
Ototoxicity	VP-16	0.542	2.582	0.020
Dermatological disorders	VP-16	0.478	2.177	0.045
Nephrotoxicity	5-FU	0.686	3.771	0.002
Neurotoxicity	Taxol	0.542	2.582	0.020
Nephrotoxicity	Taxol	0.686	3.771	0.002
Neurotoxicity	Paclitaxel	0.542	2.582	0.020
Nephrotoxicity	Paclitaxel	0.686	3.771	0.002
Constitutional symptoms	Radiotherapy	−0.620	−3.162	0.006
Head and neck tumours				
Neurotoxicity	Daily dose cisplatin	0.379	2.089	0.047
Neurotoxicity	Dexamethasone	0.556	3.407	0.002
Constitutional symptoms	Radiotherapy	−0.466	−2.687	0.012
Lung tumours				
Dermatological disorders	Daily dose cisplatin	0.282	2.120	0.039
Neurotoxicity	Dexamethasone	0.312	2.368	0.022
Nephrotoxicity	Dexamethasone	−0.316	−2.404	0.020
Nephrotoxicity	Radiotherapy	0.437	3.504	0.001
Gastrointestinal disorders	Cumulative dose cisplatin	0.483	3.982	0.000
Haematological toxicity	Cumulative dose cisplatin	0.487	4.024	0.000
Number of adverse effects	Cumulative dose cisplatin	0.484	3.990	0.000
Melanoma				
Constitutional symptoms	Daily dose cisplatin	−0.889	−3.354	0.044
Number of adverse effects	Radiotherapy	0.913	3.873	0.030

Spearman non-parametric correlation between adverse effects and chemotherapy protocols. SpR, Spearman R; P-value (P<0.05 significant, P<0.001 highly significant).

**Table IV:** Correlation of side effects.

		SpR	t(N-2)	P-value
Sleep-wake disorders	Dermatological disorders	−0.193	−2.168	0.032
Haematological toxicity	Dermatological disorders	0.236	2.676	0.008
Respiratory disorders	Gastrointestinal disorders	0.222	2.499	0.014
Ototoxicity	Gastrointestinal disorders	0.184	2.058	0.042
Ototoxicity	Genital apparatus disorders	0.197	2.210	0.029
Hepatic toxicity	Genital apparatus disorders	0.185	2.070	0.041

Spearman non-parametric correlation among adverse effects. SpR, Spearman R; P-value (P<0.05 significant, P<0.001 highly significant).
